# Milk-Derived Extracellular Vesicles: A Novel Perspective on Comparative Therapeutics and Targeted Nanocarrier Application

**DOI:** 10.3390/vaccines12111282

**Published:** 2024-11-15

**Authors:** Muttiah Barathan, Sook Luan Ng, Yogeswaran Lokanathan, Min Hwei Ng, Jia Xian Law

**Affiliations:** 1 Department of Tissue Engineering and Regenerative Medicine, Faculty of Medicine, Universiti Kebangsaan Malaysia, Cheras, Kuala Lumpur 56000, Malaysia; lyoges@ppukm.ukm.edu.my (Y.L.); angela@ppukm.ukm.edu.my (M.H.N.); 2 Department of Craniofacial Diagnostics and Biosciences, Faculty of Dentistry, Universiti Kebangsaan Malaysia, Jalan Raja Muda Abdul Aziz, Kuala Lumpur 50300, Malaysia; ngsookluan@ukm.edu.my

**Keywords:** milk, extracellular vesicles, therapeutics, drug delivery, vaccines, nanocarriers

## Abstract

Milk-derived extracellular vesicles (mEVs) are emerging as promising therapeutic candidates due to their unique properties and versatile functions. These vesicles play a crucial role in immunomodulation by influencing macrophage differentiation and cytokine production, potentially aiding in the treatment of conditions such as bone loss, fibrosis, and cancer. mEVs also have the capacity to modulate gut microbiota composition, which may alleviate the symptoms of inflammatory bowel diseases and promote intestinal barrier integrity. Their potential as drug delivery vehicles is significant, enhancing the stability, solubility, and bioavailability of anticancer agents while supporting wound healing and reducing inflammation. Additionally, bovine mEVs exhibit anti-aging properties and protect skin cells from UV damage. As vaccine platforms, mEVs offer advantages including biocompatibility, antigen protection, and the ability to elicit robust immune responses through targeted delivery to specific immune cells. Despite these promising applications, challenges persist, including their complex roles in cancer, effective antigen loading, regulatory hurdles, and the need for standardized production methods. Achieving high targeting specificity and understanding the long-term effects of mEV-based therapies are essential for clinical translation. Ongoing research aims to optimize mEV production methods, enhance targeting capabilities, and conduct rigorous preclinical and clinical studies. By addressing these challenges, mEVs hold the potential to revolutionize vaccine development and targeted drug delivery, ultimately improving therapeutic outcomes across various medical fields.

## 1. Introduction

### 1.1. Extracellular Vesicles

Extracellular vesicles (EVs) are a diverse group of membrane-bound vesicles that originate from both eukaryotic and prokaryotic cells [1]. These vesicles are released into the extracellular space, playing a pivotal role in intercellular communication by transporting a variety of biological molecules, such as messenger RNA (mRNA), microRNA (miRNA), long noncoding RNA (lncRNA), circular RNA (circRNA), DNA, proteins, lipids, and bioactive molecules (cytokines and growth factor) [2,3]. They are characterized by their complex lipid bilayers, which are enriched with cholesterol, sphingolipids, and phospholipids that contribute to EV integrity, membrane fluidity, and functionality [4]. In addition, lipid bilayers help EVs protect their cargo from enzymatic degradation, ensuring the safe delivery of these molecules [5].

Among the EV subtypes, exosomes were initially viewed as mere cellular cast-offs when discovered in 1983 [6]. However, their significance transformed dramatically in 1996 when Raposo et al. uncovered their surprising role in immune cell communication [7]. The plot further thickened in 2007 when Valadi et al. revealed that exosomes act as postal workers, carrying crucial messages, specifically, those of mRNA and miRNA, between cells [8]. This breakthrough sparked extensive research into the roles of exosomes and their RNA cargo, particularly miRNAs, in cellular communication and regulation [3]. As a fundamental aspect of cellular biology, EVs, including exosomes, are produced and released by cells across all domains of life, with variations in their morphology, biogenesis, composition, and functional roles [7].

**Figure 1:**
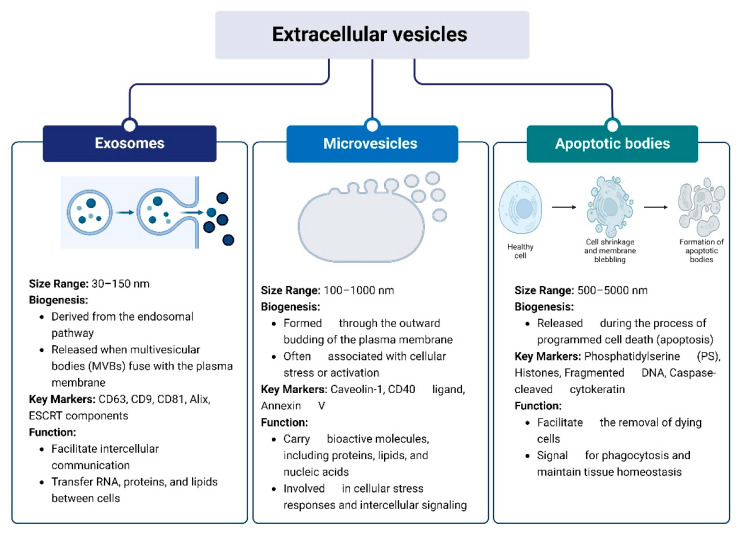
The different types of extracellular vesicles based on their size, origin, and biogenesis pathways.

The minimal information for studies of extracellular vesicles (MISEV) guidelines (2023) provide a comprehensive framework for classifying EVs into distinct subtypes based on their biogenesis, size, and composition [9]. These guidelines identify three primary categories of EVs: exosomes, microvesicles (MVs)/microparticle/ectosomes, and apoptotic bodies, each with unique characteristics and functions [9]. Figure 1 demonstrates the classification of EVs. Exosomes, typically measuring between 30 and 150 nm in diameter, are derived from the endosomal pathway and are released into the extracellular space when multivesicular bodies fuse with the plasma membrane [10,11]. This process distinguishes exosomes from other types of EVs, as their formation is tightly linked to the cell’s endosomal sorting complex. Key markers for exosomes include CD63, CD9, CD81, Alix, and components of the endosomal sorting complex required for transport (ESCRT) [12]. Microvesicles, on the other hand, are generally larger, ranging from 100 to 1000 nm, and are formed through the outward budding of the plasma membrane. This budding process is often associated with cellular stress or activation, leading to the release of MVs that carry a variety of bioactive molecules, including proteins, lipids, and nucleic acids [13]. They are characterized by markers such as caveolin-1, CD40 ligand, and annexin V. Their release is often regulated by cytoskeletal reorganization in response to increased intracellular calcium levels [14]. Lastly, apoptotic bodies, which are the largest among the three, range from 500 to 5000 nm in size and are released during the process of programmed cell death (apoptosis) [15]. These vesicles play a key role in the removal of dying cells and are characterized by several distinct markers. Phosphatidylserine (PS) is a prominent marker that becomes exposed on the outer membrane of apoptotic bodies, signaling for phagocytosis. This exposure is often detected using annexin V, a protein that binds specifically to PS [16]. In addition to PS, apoptotic bodies frequently contain histones and fragmented DNA, reflecting the breakdown of nuclear material during apoptosis. Caspase-cleaved cytokeratin, a result of cytoskeletal breakdown, is another marker associated with these vesicles [17]. These markers help distinguish apoptotic bodies from other EVs, highlighting their role in cellular clearance and maintaining tissue homeostasis. Each type of EV plays distinct roles in cellular communication and physiological processes, contributing to intercellular signaling and the regulation of various biological functions [18]. The MISEV guidelines emphasize the importance of using a combination of advanced techniques, such as electron microscopy, nanoparticle tracking analysis (NTA), and immunoblotting, to accurately identify and characterize these EV subtypes [19]. This approach ensures that studies on EVs are conducted with a high degree of precision, thereby facilitating a better understanding of their roles in intercellular communication, disease progression, and potential therapeutic applications [3].

In recent years, EVs have garnered significant attention from researchers owing to their involvement in diverse pathophysiological processes and their potential as biomarkers and therapeutic targets [20]. With an abundance of information encapsulated within their lipid bilayers, EVs are ubiquitous in various biological fluids, including blood, pleural fluid, and urine, rendering them amenable for biomarker exploration and clinical utility [21]. Animal-derived EVs refer to EVs that are obtained from various animal-derived sources such as milk, blood, and saliva, and have gained attention for their potential applications in various fields, including medicine and agriculture [22,23,24,25]. These EVs are released by cells present in animal tissues and fluids, carrying a diverse cargo of proteins, nucleic acids, lipids, and other bioactive molecules. Animal-derived EVs have gained significant attention in biomedical research due to their potential therapeutic applications and diagnostic utility [26,27]. Investigations into animal-derived EVs have revealed therapeutic potential, encompassing antioxidant, antibacterial, anti-inflammatory, anticancer, and regenerative properties akin to mammalian EVs.

The novelty of this review highlights the therapeutic potential of milk-derived EVs (mEVs) across various applications, including immunomodulation, gut microbiota modulation, and cancer treatment. Notably, it emphasizes the unique properties of mEVs derived from different animal sources, such as cows and camels, which can influence their biological functions and therapeutic efficacy. Key strategies to enhance mEV targeting include surface modifications and the incorporation of nanoparticles, which improve specificity for tumor cells. Furthermore, mEVs show promise in vaccine development, particularly for oral and cancer vaccines, enhancing immune responses. However, challenges such as isolation, purification, and targeting specificity remain significant hurdles in clinical translation. This comprehensive analysis underscores the need for continued research to address these challenges and optimize mEV applications in therapeutic settings.

### 1.2. Milk-Derived Extracellular Vesicles (mEVs)

Milk is a complex fluid with a dynamic composition influenced by various factors such as species, breed, diet, and lactation stage. Milk components are organized into three main phases: fat dispersed in an “oil-in-water” emulsion forming milk fat globules (MFG), caseins organized into micelles, and whey proteins [28,29,30]. Milk is a crucial source of nutrition for newborn mammals, providing essential nutrients and bioactive components vital for their development and health. Beyond its nutritive elements, milk plays a critical role in bolstering the newborn’s immune system development [31]. Recent research has delved into the molecular mechanisms underlying the transfer of maternal messages to offspring through milk, highlighting the presence of anti-infective and anti-inflammatory components such as oligosaccharides, lactoferrin, lysozyme, α-lactalbumin (α-La), and immunoglobulins [32,33]. Furthermore, milk contains transcription modulatory elements such as small RNAs, found in different milk fractions either as free molecules or packaged in vesicles [34].

Milk-derived EVs (mEVs) can be isolated from various milk phases, predominantly from the whey fraction. The EVs can be distinguished based on their source within the milk and their association with different milk components [35]. They have been studied extensively due to their abundance and stability in milk [36]. These vesicles exhibit remarkable stability, withstanding harsh conditions such as low pH, boiling, and freezing, which suggests their potential to survive passage through the gastrointestinal tract (GIT) [23]. The resistance of mEVs to gastric digestion is a critical factor that enhances their ability to deliver bioactive molecules to the intestine, where they can exert significant biological activities [37]. The mEVs, first identified in 1971, have been extensively studied since 2007, particularly in human colostrum and breast milk, revealing their immunological significance [25]. Subsequent research has confirmed the presence of mEVs in various mammals, including cows [38], camels [39], buffaloes [40], porcine [41], horses [42], sheep [38], and giant pandas [43]. A study has found that EVs from bovine milk contain over 16,000 mRNAs and more than 800 miRNAs [44]. Notably, 176 known and 315 novel mature miRNAs have been identified in porcine mEVs, while bovine colostrum shows elevated levels of immune-related miRNAs [45], with other non-coding RNAs like lncRNAs, circRNAs, and transfer RNAs (tRNAs) also identified [46]. A comprehensive RNA sequencing analysis identified 2466 novel lncRNAs, 809 annotated lncRNAs, and 61 circRNAs in mEV derived from porcine. These lncRNAs share similar characteristics with those found in other mammals but are expressed at higher levels than mRNAs [47]. These RNAs are involved in immune regulation, neurodevelopment, and reproduction [44]. Bovine mEVs also have a rich protein content, with over 2100 proteins detected, many of which play roles in inflammation, cell growth, and gut health [48]. Additionally, 395 lipids have been identified in these EVs, but no relevant studies on DNA content have been reported [49].

The mEVs are challenging to characterize due to the lack of standardized methods for milk pre-processing, storage, and exosome isolation [50]. Several techniques are currently available for separating exosomes from milk, including ultracentrifugation, size exclusion chromatography (SEC), and density gradient centrifugation (DGC) [51]. Ultracentrifugation is the most common method for isolating exosomes from milk [52]. It involves centrifuging raw milk to remove fat globules and debris, followed by precipitating exosomes at high speeds [53]. While this technique offers a relatively high yield and scalability, it has drawbacks, including lower purity compared to other methods, as it can co-pellet proteins, lipids, and other non-exosomal components. Additionally, it can be harsh on exosomes, potentially affecting their integrity and bioactivity [52]. This method is best suited for general exosome isolation in initial studies or large-scale extractions, but for therapeutic or biomarker development, additional purification steps may be required to enhance purity [54]. SEC is best suited for applications where preserving the structural integrity and bioactivity of exosomes is crucial, such as in functional studies, therapeutic development, or biomarker discovery [55]. The method is gentle, ensuring that the exosomes remain intact and functional, and is highly reproducible [56]. It can also be easily combined with other techniques. However, SEC typically yields lower quantities compared to ultracentrifugation and may result in some contamination with similarly sized particles [57]. It is particularly ideal for applications requiring high purity, including functional assays, therapeutic use, and RNA/DNA analysis [58]. DGC is best suited for high-purity exosome isolation where the removal of non-vesicular particles is critical, such as in high-precision research or clinical applications [59]. This method provides the highest purity by separating exosomes based on their buoyant density, effectively removing contaminants like proteins and lipoproteins. It is particularly effective for detailed proteomics or transcriptomics studies [52]. However, DGC is time-consuming, labor-intensive, and may yield lower quantities compared to ultracentrifugation. It requires precise handling to set up the density gradient. Overall, DGC is ideal for clinical-grade isolation or when the high purity of exosomes is essential [60]. Additionally, polymer-based precipitation is a fast and scalable method suitable for preliminary exosome isolation but often results in lower purity [54]. Immunoaffinity capture provides high specificity by targeting exosome surface proteins, making it ideal for diagnostic or therapeutic applications, though it is costly and may yield lower quantities if markers are under expressed [61]. Microfluidic devices offer high purity and sensitivity with minimal sample volumes, useful for point-of-care diagnostics, but face scalability challenges. Filtration and ultrafiltration are cost-effective for pre-enrichment but may affect exosome integrity [62], while tangential flow filtration (TFF) is effective for large-scale isolation without clogging [63]. Emerging techniques like aqueous two-phase systems (ATPS) and acoustic nanofiltration show promise for high-yield and gentle isolation, respectively [64], but are still under exploration. Additional methods such as field flow fractionation (FFF), hydrostatic filtration dialysis (HFD), magnetic-activated cell sorting (MACS), and electrophoresis-based methods provide flexibility for various isolation needs based on size, charge, or specificity [65,66]. Exosomes are commonly characterized using NTA, transmission electron microscopy (TEM), Western blot (WB), and flow cytometry. NTA and TEM analyze size distribution and morphology of exosomes, respectively. WB and flow cytometry verifies milk exosomal markers such as CD9/63/81, Tsg101, HSP70, Alix, and flotillin 1 [67]. Cellular uptake was normally performed using the fluorescently tagged exosomes [68,69]. These methods and markers provide comprehensive insights into the isolation and identification of milk-derived exosomes, facilitating further research into their biological functions and potential applications.

## 2. Functions of mEVs

### 2.1. Immunomodulation

The immunomodulatory effects of mEVs extend to modulating macrophage differentiation, cytokine production, and inflammatory outcomes, exhibiting therapeutic potential in diverse disorders such as bone loss, fibrosis, and cancer [70]. The presence of immunoregulatory miRNAs and transforming growth factor-beta (TGF-β) in mEVs underscores their immunoregulatory potential, implicated in modulating the immune response, including the induction of regulatory T cells (Tregs) and differentiation of T helper 17 (Th17) cells [71]. While Tregs play a beneficial role in immune regulation, promoting tolerance and preventing autoimmunity, Th17 cells can contribute to inflammation and autoimmune diseases when dysregulated [72]. The ability of mEVs to induce Th17 cell differentiation suggests a dual role in health and disease. In healthy individuals, mEVs may promote immune tolerance and regulatory responses. However, under conditions of inflammation or autoimmune disease, they could exacerbate pathogenic Th17 cell differentiation, potentially contributing to disease pathogenesis. Through these mechanisms, mEVs contribute to the growth, development, and overall health of neonates, highlighting their significance in early life physiology and potential applications in health and disease [36]. A study has showed that the mEVs could act as immunomodulators, showing their ability to induce CD69 expression and enhance IFN-γ production in NK cells and γδ T cells. These findings contribute to the understanding of how dietary factors can influence immune cell function, offering insights that may inform the development of novel immunotherapeutic strategies [73].

### 2.2. Modulation of Gut Microbiota

A study has examined the impact of mEVs on gut microbiota and blood metabolites in C57BL/6 female and male mice, whereby analysis observed that mEVs increased levels of beneficial microbes such as *Akkermansia*, *Muribaculum*, and *Turicibacter* while reducing harmful bacteria like *Desulfovibrio*. In blood metabolites, mEVs induced changes in lipid and amino acid metabolism, elevating anti-inflammatory factors and enriching pathways related to immune function and metabolism. Overall, these findings suggest that mEVs may offer health benefits by modulating gut microbiota and blood metabolites without promoting harmful bacterial colonization, hinting at their potential as safe bioactive molecules with protective effects against inflammatory and metabolic diseases [74]. Furthermore, the oral administration of bovine mEVs has shown promise in modulating gut microbiota, alleviating symptoms of inflammatory bowel disease (IBD), and promoting intestinal barrier integrity by enhancing mucin secretion [75]. In addition, a study explored the impact of mEVs on gut microbiota and intestinal immunity in mice. Analysis reveals that mEVs alter gut microbiota composition and modulate short-chain fatty acids (SCFAs). Moreover, mEVs enhance the expression of genes crucial for intestinal barrier integrity (Muc2, RegIIIγ, Myd88, and GATA4) and increase IgA levels, which are vital for mucus layer maintenance. These findings highlight mEVs’ potential in shaping gut health and immune regulation [76,77].

### 2.3. Anticancer Effect

mEVs are promising candidates for drug delivery due to their bilipid membrane structure, which is similar to liposomes and enables them to carry both hydrophilic and lipophilic drugs [78]. Methods such as incubation and electroporation can be employed to load drugs into or onto exosomes, potentially enhancing their therapeutic effects. mEVs are noted for their controlled release, suggesting increased stability and targeted delivery to specific sites. They have been investigated as carriers for various drugs and biologics, including anticancer agents. For example, one study demonstrated that goat mEVs efficiently delivered the photosensitizer chlorin e6 (Ce6) into tumor cells, achieving the best tumor inhibition rate and longest survival in tumor-bearing mice [79]. Research has also focused on the application of milk-derived exosomes (mEXOs) in nanomedicine, particularly as enhancers of anticancer therapy. A pioneering study used bovine mEXOs as carriers for chemopreventive and chemotherapeutic drugs, showing enhanced antiproliferative effects in cancer cell lines and improved antitumor activity in mouse models compared to free drugs [80]. These loaded mEXOs improved the stability, solubility, and bioavailability of the drugs compared to their free forms [81]. Moreover, the exosomal encapsulation of these agents enhanced their antitumor activity in preclinical models, underscoring the potential of mEXOs for delivering various anticancer agents. For instance, paclitaxel-loaded mEXOs administered orally showed significant tumor growth inhibition in lung cancer xenografts with reduced systemic and immunologic adverse effects [82]. An in vitro study showed that doxorubicin-loaded mEXOs, combined with hyaluronic acid, induced tumor cell death, indicating their potential for targeted cancer therapy [83]. Additionally, Zhang et al. demonstrated that a drug delivery system based on mEXOs conjugated to doxorubicin (Dox) via a pH-cleavable bond exhibited controlled drug release and biocompatibility, making it effective in treating oral squamous cell carcinomas [84]. Furthermore, mEXOs loaded with celastrol or anthocyanidins displayed enhanced anticancer effects and good tolerability in vivo [85]. mEXOs have demonstrated potential as carriers for various anticancer agents, such as curcumin, anthocyanidin, and siRNA, enhancing their stability, bioavailability, and therapeutic efficacy. Exosomes protect and deliver siRNA to target cells efficiently because they are resistant to RNase enzymes that degrade siRNA. Research has successfully used mEXOs to silence specific cancer genes (e.g., VEGF, EGFR, AKT, MAPK, KRAS, SUR, and BCL2) in vitro [86]. Additionally, mEXOs have been employed to deliver miR-148a-3p to liver and colon cancer cells, leading to the reduced expression of genes like AKR1C1, AKR1C2, CYP3A5, CAB39L, ODAM, and NEGR1 [87,88].

While research has primarily focused on cow mEVs, exploring mEXOs from other *Bovidae* species could reveal whether these anticancer effects are conserved [89]. Studies on goat mEXOs have shown efficient internalization by macrophages and their potential for detecting inflammatory processes. High doses of goat mEXOs can enhance macrophage activation and induce pro-inflammatory cytokines, potentially suppressing tumor growth and metastasis by polarizing macrophages to an M1-like phenotype [90]. Similarly, buffalo mEXOs have demonstrated potent antitumor effects through proteins such as Rabs, CHMPS, VPS, butyrophillin, xanthine dehydrogenase, lactadherin, and adipophilin, inducing significant cell death in colon cancer cells compared to other mEXOs [91]. Camel mEXOs have been found to induce apoptosis in HepG2 and CaCo2 cells, with increased Bax expression, enhanced caspase 3 activity, and decreased Bcl2 expression, while sparing normal Vero cells, indicating selective anticancer effects [92]. Furthermore, yak mEXOs have shown the ability to promote survival of IEC-6 cells under hypoxic conditions by influencing oxygen-sensitive prolyl hydroxylase (PHD)-1 and inhibiting hypoxia-inducible factor (HIF)-α and its downstream target VEGF, suggesting better hypoxia tolerance compared to cow mEXOs [93]. Among exosomes isolated from various lactation periods, those derived from colostrum exhibit the highest apoptotic activity on HepaRG cells, characterized by increased DNA damage, the upregulation of pro-apoptotic markers (Bax and caspase 3), and decreased anti-apoptotic Bcl2. Colostrum-derived exosomes also reduce inflammation-related genes (TNFα, NFkB, TGFβ1, and Cox2) and the angiogenesis-related gene VEGF, compared to exosomes from other lactation periods. Additionally, they show significantly higher levels of lactoferrin and kappa casein, suggesting a more potent anti-cancer effect [94].

### 2.4. Wound Healing

mEXOs have also shown promise in supporting wound healing through drug and biologic delivery. For instance, encapsulating miR-31-5p in mEXOs significantly accelerated wound healing in diabetic mice, improved endothelial cell function, and stimulated angiogenesis. This was attributed to the enhanced cellular uptake and stability of miR-31-5p when delivered via mEXOs [95]. Additionally, diabetic wound healing is often hindered by oxidative stress and impaired Nrf2 signaling. Loading mEXOs with siRNA targeting Keap1 (a Nrf2 repressor) using ultrasound led to reduced oxidative stress, enhanced wound healing, increased collagen formation, and better neovascularization in a diabetic mouse model, highlighting mEXOs’ potential as a scalable and cost-effective delivery system for RNA therapies [96]. Furthermore, mEXOs have been found to play a role in scar-free wound healing by modulating the TGF-β/Smad signaling pathway. They suppress cell migration and increase the expression of transforming growth factor beta-3 (TGFβ3), which is associated with scar reduction. The enhanced phosphorylation of Smad3 by mEXOs promotes improved cell growth and extracellular matrix (ECM) formation, suggesting their potential in minimizing scars from skin damage and surgical incisions [97].

### 2.5. Inflammation

Research into mEVs has revealed their potential role in inflammation and immune modulation. Proteins such as S100A8/A9 within mEVs have been shown to enhance pro-inflammatory responses by activating the NF-κB pathway, particularly in inflammatory diseases. These proteins are involved in defense responses, neutrophil degranulation, and antimicrobial peptides, highlighting their role in innate immune responses in cows with inflammatory conditions [98]. In comparative studies, sheep mEV miRNAs were analyzed using next-generation sequencing alongside cow mEXO miRNAs. Key miRNAs identified include miR-148a, let-7b, miR-21, miR-191, oar-miR-125b, and miR-27a, which are crucial in regulating B cell tolerance, inflammatory responses, T-cell biology, and innate immune responses. Pathway analysis of the target genes of these highly expressed sheep mEV-miRNAs revealed their involvement in protein processing, endoplasmic reticulum function, ubiquitin-mediated proteolysis, cell adhesion molecules, phospholipase D signaling, and glycerophospholipid metabolism, all critical for protein metabolism, EV structure, and lipid metabolism [99]. Further studies showed that mEV treatment at higher concentrations can modulate the expression of pro-inflammatory cytokines and improve intestinal barrier function. Specifically, mEV treatment reduced pro-inflammatory cytokines such as IL-8, TNF-α, IL-1β, IL-12, and IL-17, while promoting anti-inflammatory cytokines like IL-10. Additionally, mEVs influenced key genes involved in intestinal barrier function, such as MMP-9 [100]. Research on goat mEVs demonstrated their ability to regulate immune cells and restore intestinal barrier integrity during IBD. An increase in IL-8 gene expression and protein release was observed 48 h post-treatment, indicating the complex modulation of inflammatory mediators by mEVs [101]. Treatment of porcine intestinal epithelial cells with goat mEVs following exposure to LPS or H_2_O_2_ resulted in reduced pro-inflammatory responses, as evidenced by the decreased expression of TLRs and cytokines (CXCL8, TNFA, and NOS2), demonstrating the immunomodulatory potential of mEVs [102].

### 2.6. Skin Health

Research indicates that bovine mEXOs positively impact UV-induced aging and damage in skin cells [103]. The mEXOs prevent UV-induced intracellular reactive oxygen species production in keratinocytes, reduce melanin production in melanocytes, and suppress matrix metalloproteinase expression and enhance collagen production in dermal fibroblasts. These observations suggest that mEXOs have therapeutic potential for repairing UV-irradiated skin aging and damage. Another study explored the anti-aging effects of bovine mEXOs on human skin. mEXOs were found to enhance gene expression related to skin moisturization in keratinocytes and fibroblasts in vitro. Furthermore, mEXOs promoted fibroblast migration and restored collagen expression post-UV exposure. Finally, in a 28-day study on 31 female volunteers, mEXOs demonstrated efficacy in preserving moisture and reducing wrinkles. These findings highlight bovine mEXOs as promising skincare agents with antiaging properties [104].

### 2.7. Bone Regeneration

Bioactive components in milk, including proteins and miRNAs, significantly impact bone metabolism. Studies have shown that bovine mEXOs can reduce bone loss and promote osteoclast differentiation in vitro. In vivo, mEXOs have demonstrated beneficial effects on osteoporosis in animal models, suggesting their potential for treating bone-related diseases [105]. Milk-derived small extracellular vesicles (mSEVs) are proposed as effective carriers for enhancing the efficacy of icariin (ICA), a drug used to treat inflammatory bone defects, due to their biocompatibility and low immunogenicity. mSEV-ICA has shown enhanced osteogenic effects on MC3T3-E1 cells and in a mouse skull defect model compared to free ICA. This improvement is likely due to the enhanced stability and increased uptake rate of mSEVs. Bioinformatics analysis revealed an enrichment of bone-related genes in the mSEV-ICA group, with GJA1 identified as a key regulator of bone growth. Further research suggested that the transcription factor STAT5a plays a significant role in the regulatory effects of mSEV-ICA on GJA1 expression [106].

### 2.8. Hepatoprotective and Liver Regeneration

The oral administration of bovine mEVs has been shown to enhance gut barrier integrity by reducing intestinal inflammation, improving epithelial tight junctions, and promoting mucus secretion [107]. mEV treatment not only alleviates colonic inflammation but also mitigates liver pathology associated with non-alcoholic steatohepatitis (NASH). These protective effects are attributed to specific miRNAs, such as miR-148a, which are linked to tight junctions and gut barrier function, as well as the activation of the AMPK pathway. Additionally, mEVs interact with lamina propria immune cells in the colon, facilitating their transition to an immunosuppressive phenotype and reducing pro-inflammatory cytokine production. This makes mEVs a promising natural, edible nanoparticle-based therapy for gut-related disorders, particularly advantageous for individuals with lactose intolerance or inflammatory bowel disease symptoms exacerbated by lactose or long-chain triacylglycerols [107]. Furthermore, mEXOs encapsulated with forsythiaside A (FA) and modified with hyaluronic acid (HA) have shown improved delivery of drug-loaded exosomes to target cells. This modification facilitates specific interaction with CD44 receptors, enhancing the anti-liver fibrosis effects of FA by inhibiting NLRP3-mediated pyrotosis. This was evidenced by reduced NLRP3 production in TAA-induced liver fibrosis models treated with HA-mExo-FA [108].

### 2.9. Antibacterial Effect

The camel mEXOs exhibited bacteriostatic effects, particularly against Gram-negative bacteria such as *Escherichia coli*, *Pseudomonas aeruginosa*, and *Proteus mirabilis*, as well as fungistatic effects against *Candida albicans*. However, they did not exhibit bactericidal effects against Gram-positive bacteria such as *Staphylococcus aureus*, *Micrococcus luteus*, and *Enterococcus faecalis* [92]. A study found that bovine mEXOs functionalized with a combination therapy of isobavachalcone (IS) and polymyxin B (PB) as IP-EXOs showed significant antibacterial efficacy against multidrug-resistant pathogens, with nearly 100% microbial inhibition in fresh orange juice and accelerated wound healing in mouse models. IP-Exo holds promise for use in the food industry and animal husbandry as a defense against bacterial pathogens [109,110].

### 2.10. Antiviral Effect

Goat mEXOs significantly decreased the infectivity of dengue and reduced its replication and secretion of mature virions. Heat inactivation of goat mEXOs did not affect its antiviral properties against dengue, indicating that the exosomes themselves were responsible for the inhibition. Furthermore, the treatment of goat mEXOs with RNase abolished its antiviral effects, further confirming the direct role of exosomes in inhibiting dengue. Additionally, the particular exosome inhibited the infectivity of Newcastle disease virus strain Komarov, but not human immunodeficiency virus, suggesting that the antiviral activity of exosome may be specific to certain viruses [111].

Overall, these findings underscore the numerous advantages of mEXOs, which are multifaceted and increasingly recognized as pivotal in shaping host health and physiology, including minimal adverse immune and inflammatory responses, highlighting their promising potential for targeted disease treatment through clinical drug delivery systems. Table 1 summarizes the potential therapeutic applications of mEVs based on recent research findings meanwhile Table 2 shows the use of mEVs derived from various animal as drug carriers and their therapeutic effects when used alone.

## 3. Challenges and Future Direction in Translation of mEV-Based Therapies

The translation of mEV-based therapies presents both significant challenges and promising opportunities for therapeutic advancement. A key limitation lies in the lack of specificity of mEVs to target recipient cells, which can reduce their efficacy in targeted treatments. Surface modifications, such as attaching ligands like hyaluronan or folic acid, have been shown to enhance tissue retention and targeting capabilities via receptor-mediated endocytosis, particularly in cancer therapy [112]. For example, HA-coated bovine mEXOs exhibited improved tumor targeting and antitumor efficacy compared to unmodified mEXOs [113]. However, the variability in mEV composition, influenced by factors such as the donor’s immune status, lactation stage, diet, and environmental stressors, presents an additional challenge. These factors can significantly alter the bioactive content of mEVs, thereby impacting their therapeutic potential and consistency [114,115].

Moreover, current isolation methods, including ultracentrifugation and SEC, are labor-intensive and not scalable, limiting large-scale production. To address this, strategies such as optimizing the cell culture environment using three-dimensional (3D) systems and exploring novel production methods like sonication and nitrogen cavitation could enhance both the yield and efficiency of mEV production [116,117]. Another critical challenge revolves around safety concerns, particularly regarding immunogenicity and off-target effects. Preclinical studies must rigorously assess the biodistribution, pharmacokinetics, and toxicity of mEV formulations in animal models before clinical trials. This highlights the urgent need for standardized guidelines to facilitate regulatory approval and ensure the safe clinical use of mEVs [118].

The lack of standardized protocols for loading therapeutic cargo into mEVs and for surface modifications to enhance targeting specificity further impedes the development of therapeutic formulations. Optimizing strategies for efficiently loading mEVs with drugs, nucleic acids, or proteins while maintaining their structural integrity and biological function is crucial [119]. Nevertheless, mEVs present exciting opportunities, particularly in neuroprotective therapies for neurodegenerative diseases such as Parkinson’s disease. mEVs have been used to deliver bioactive compounds, like epicatechin gallate, which exhibit improved stability, bioavailability, and lower immunogenicity compared to synthetic nanoparticles [120,121]. Their biocompatibility and low systemic toxicity add to their potential as versatile therapeutic agents, with broad applications in fields like oncology, immunology, and regenerative medicine.

Looking forward, future research on animal-derived EVs will likely focus on elucidating the molecular cargo of EVs from various animal sources, such as proteins, nucleic acids, lipids, and other bioactive molecules, to fully understand their roles in intercellular communication and disease modulation [51]. Enhanced profiling will enable researchers to better harness EVs’ therapeutic potential. Additionally, improving the therapeutic efficacy and specificity of EVs through targeted modifications and optimizing the loading of therapeutic agents will be critical for advancing clinical applications [122,123,124]. Research into the immunomodulatory properties of mEVs is also emerging as a promising avenue, with potential applications in treating autoimmune diseases, inflammatory conditions, and cancer [67,125].

Advancing isolation and purification techniques, along with developing standardized protocols, will be essential to enhance the scalability, reproducibility, and clinical translation of mEV-based therapies [126,127]. Finally, rigorous preclinical and clinical studies are needed to assess the safety, efficacy, and long-term impacts of these therapies, with a focus on evaluating biodistribution, pharmacokinetics, and toxicity profiles in both animal models and human trials [128,129]. With continued interdisciplinary collaboration and technological innovation, the field of animal-derived EV research holds great promise for revolutionizing therapeutic interventions across various biomedical applications.

## 4. mEVs as Targeted Nanocarriers

mEVs possess inherent advantages in terms of safety and compatibility, but their natural targeting capabilities are often limited. This intrinsic limitation has driven the development of several innovative strategies aimed at enhancing the specificity, efficacy, and delivery potential of mEVs to targeted cells and tissues, especially in therapeutic applications such as cancer treatment, gene therapy, and immunomodulation. A recent study has demonstrated the successful modification of mEXO surfaces with folate-conjugated lipids. This approach significantly enhances the exosomes’ ability to selectively target folate receptor-positive tumor cells, thereby improving the efficiency of doxorubicin delivery. The enhanced targeting capabilities have led to increased drug uptake in cancer cells, highlighting the potential of surface modifications to optimize therapeutic outcomes [130].

A promising technique to enhance mEV targeting involves the incorporation of superparamagnetic iron oxide nanoparticles (SPIONs). These nanoparticles can be loaded into mEVs through methods such as electroporation or natural incubation, giving the vesicles magnetic properties. When used in conjunction with external magnetic fields, SPION-loaded mEVs can be guided and accumulated at specific sites within the body, such as tumor tissues. Moreover, SPIONs can be modified with targeting ligands, which improves the binding efficiency of the mEVs to target cells. This magnetic navigation technique is particularly advantageous for improving the penetration of mEVs in tissues with poor vascularization, such as solid tumors, thereby enhancing the delivery of therapeutics [131,132].

Glycoengineering, which involves the genetic modification of the glycosylation patterns on the surface of mEVs, has emerged as a sophisticated approach to enhance cell-specific targeting. By altering the glycan structures on mEV surfaces, researchers can guide these vesicles towards specific cell types, such as endothelial cells or dendritic cells. This method leverages the glycocalyx, a sugar-rich outer layer of the vesicles, to facilitate selective interaction with receptors on the surface of target cells. Glycoengineering represents a highly specific targeting strategy that could revolutionize the use of mEVs in personalized medicine by allowing for precise delivery to desired tissues while minimizing off-target effects [133,134]. The introduction of polyethylene glycol (PEG) onto the surface of mEXOs has proven beneficial in improving their mucus penetrability and stability in acidic environments. This PEGylation technique facilitates the oral delivery of siRNA by protecting the nucleic acids from degradation and enhancing their bioavailability. Furthermore, PEGylation prolongs the circulation time of mEXOs in the bloodstream by reducing their clearance, thereby enhancing their overall efficacy in drug delivery applications [135].

Another approach involves ligand modification, where targeting ligands such as specific peptides or antibodies are strategically attached to the surfaces of mEXOs, enabling selective binding to overexpressed receptors on tumor cells. This targeted approach has shown to enhance drug uptake, particularly in cancer therapies, significantly increasing the efficacy of the drug delivery systems employed. By leveraging the natural targeting mechanisms of mEXOs, researchers can improve the precision of drug delivery and minimize off-target effects [136]. Targeted cancer therapy utilizing mEXOs, particularly those coated with hyaluronic acid (HA-mEXOs), shows promise in minimizing side effects and enhancing drug delivery. HA binds to CD44 receptors, which are overexpressed in tumor cells, facilitating the delivery of miR-204-5p mimics and demonstrating increased antitumor efficacy without systemic toxicity [137]. Folate receptors (FR), overexpressed in non-small cell lung cancer (NSCLC) and lung adeno-carcinoma, serve as another effective target. Bovine mEXOs conjugated with folic acid and loaded with apherin A or paclitaxel exhibit superior antitumor effects compared to non-modified versions [138]. Additionally, HA can be conjugated to mEXOs to selectively deliver doxorubicin, further enhancing therapeutic outcomes [139]. Additionally, bovine mEVs have successfully transferred tumor-suppressive mi-croRNAs, like miRNA-125b and miRNA-let-7, to prostate cancer cells, effectively inhibiting oncogenic pathways [140]. Techniques such as click chemistry and hydrophobic insertion are being explored to improve specificity by attaching various targeting molecules. For instance, amphipathic molecules like DSPE-PEG enable targeted delivery to sigma receptors in lung cancer.

Despite the potential of mEXOs as drug carriers, challenges related to large-scale production and effective surface modification need to be addressed for clinical application [80,141]. The engineering of biocompatible polymers onto the surfaces of mEXOs further enhances their stability and protects their therapeutic cargo. This polymer hybridization not only increases the circulation time of mEXOs in vivo but also improves their overall drug delivery efficiency. The combination of exosomes with polymers creates a robust platform that can withstand physiological challenges while effectively delivering therapeutic agents to target cells [142]. Ongoing research is dedicated to optimizing these procedures to ensure that mEXOs can be produced in sufficient quantities without compromising their structural and functional properties. Nevertheless, the potential of mEXOs as versatile and effective drug delivery systems continues to expand, with researchers actively exploring their applications in various therapeutic contexts [35]. Table 3 focuses on targeting strategies relevant to mEVs.

## 5. mEVs as Potential for Vaccine

Exosomes, small extracellular vesicles naturally secreted by a variety of cells, have emerged as promising tools in the field of cancer immunotherapy due to their ability to carry and deliver biomolecules like proteins, lipids, and nucleic acids [1,2]. For example, embryonic stem cell-derived exosomes (exosomes expressing GM-CSF) have been shown to reduce immune suppressor cells such as Tregs in lung cancer models, while enhancing the activity of CD8+ T cells and promoting long-term immunity [143]. Similarly, pancreatic cancer research also highlights the potential of exosomes in combination with chemotherapy, where exosome-based vaccines, loaded with drugs like gemcitabine, can reprogram the tumor microenvironment and enhance anti-tumor immune responses [144]. In ovarian cancer, exosomes derived from ascites fluid contain immune-activating proteins like HSP-70 and MHC-I, and they can induce T-cell activation via dendritic cells. These examples illustrate how exosomes can influence both local tumor immunity and systemic immune responses, making them a versatile tool in cancer therapy [145,146]. They have shown significant promise in cancer immunotherapy across various clinical trials, although the results remain mixed. In one phase I trial, dendritic cell-derived exosomes (DEXs) loaded with MAGE peptides were tested in non-small cell lung cancer (NSCLC) patients, leading to increased NK cell activity but limited T-cell responses, suggesting that while exosomes can activate innate immunity, their ability to trigger adaptive immune responses may depend on factors like the maturity of the dendritic cells used [147]. Another phase I trial using ascites-derived exosomes (AEXs) combined with GM-CSF for colorectal cancer patients demonstrated strong anti-tumor cytotoxic T-lymphocyte (CTL) responses against the carcinoembryonic antigen (CEA), a colorectal cancer biomarker, indicating that exosome-based vaccines could stimulate targeted immune responses [148]. Additionally, a study involving exosomes derived from mesenchymal stem cells (MSCs) for chronic kidney diseases showed no adverse effects and improved kidney function, paving the way for MSC-derived exosome therapies in non-cancer indications as well [149]. A promising trial using exosomes from dendritic cells pulsed with the SART1 biomarker for esophageal squamous cell carcinoma saw one patient remain stable for 20 months, further highlighting the potential for personalized exosome-based vaccines. These examples demonstrate the versatility and potential of exosome-based vaccines in cancer treatment, although challenges remain in optimizing their effectiveness and consistency [150].

Meanwhile, bovine mEVs have gained significant attention due to their stability, biocompatibility, and ability to carry therapeutic payloads. These properties make mEVs a versatile vehicle for the delivery of tumor-associated antigens (TAAs) and other immunotherapeutic agents, with the potential to stimulate targeted and effective immune responses against cancer [29]. Another key benefit of milk exosomes over other types of exosomes or nanoparticles is their low immunogenicity. This means they are less likely to provoke unwanted immune reactions when administered, making them safer for clinical applications. While milk-derived exosomes have not yet been widely tested in clinical trials for cancer immunotherapy, their potential in this field is promising [36].

The potential of mEVs extends into cancer vaccine development. Bovine milk-derived exosomes have been loaded with HER2 to elicit robust immune responses. This approach has demonstrated the capacity to activate cytotoxic T cells and stimulate the production of anti-tumor antibodies, showing promise for personalized cancer immunotherapy [151]. These exosomes could help activate both CD8+ cytotoxic T-cells and CD4+ helper T-cells, leading to a more robust and long-lasting immune response. Because milk exosomes naturally carry immune stimulating molecules (such as MHC molecules and co-stimulatory proteins), they could be used to enhance the effectiveness of cancer vaccines by boosting the activation of immune cells that target tumor cells [152]. This could be especially beneficial for tumors that are otherwise immunologically inert or “cold”, meaning they do not elicit strong immune responses on their own. Further, mEVs can encapsulate a variety of antigens and nucleic acids, and their lipid bilayer protects these payloads from harsh biological environments, ensuring effective delivery to immune cells. Furthermore, glycoengineering and surface modification techniques have been developed to enhance antigen presentation and ensure targeted delivery to specific immune cells, like dendritic cells, promoting stronger and more specific immune responses [153].

## 6. Exosome Based Vaccine and Gut Microbiota

Exosome-based vaccines have emerged as an innovative tool to modulate gut microbiota and enhance immune responses, offering potential benefits for both infectious diseases and inflammatory conditions [154]. Unlike traditional vaccines, exosomes, as naturally derived nanovesicles, can encapsulate and deliver bioactive molecules directly to target cells, including those in the gut microbiome [155]. For instance, plant-derived exosome-like nanoparticles (ELNs) from sources like ginger and grape have been shown to selectively target beneficial bacteria in the gut [156]. A study by Yan et al., 2024 demonstrated that ginger-derived ELNs could enhance the growth of *Lactobacillus rhamnosus,* a beneficial gut bacterium known for its immunomodulatory effects, promoting anti-inflammatory responses and improving gut barrier integrity in animal models of colitisrly, and milk-derived exosomes have been investigated for their potential in shaping gut microbiota and supporting immune health [157]. Mun et al. (2022) conducted research showing that bovine milk-derived exosomes influenced the diversity of gut microbiota in mice, increasing the abundance of beneficial microbial species such as Bifidobacterium and Lactobacillus [158]. This modulation was associated with enhanced gut barrier function and reduced inflammation, demonstrating the potential of exosomes to support gut health and immune resilience through microbiome modulation. Further, exosome-based vaccines can serve as a novel approach for managing conditions like inflammatory bowel disease (IBD). One promising example involves the use of exosomes loaded with specific antigens to induce tolerance in the gut, potentially mitigating inflammatory responses characteristic of IBD. In a study by Kim et al. (2023), plant-derived exosomes loaded with curcumin, an anti-inflammatory compound, were shown to reduce inflammation and restore gut microbial balance in mice with colitis [159]. This study highlights how exosome-based therapies may offer targeted microbiome modulation while also delivering anti-inflammatory effects directly to the gut. These examples indicate the potential of exosome-based vaccines and therapeutics not only to enhance immune responses but also to reshape gut microbiota, paving the way for innovative treatments in gut-related diseases and overall immune health [160].

The interplay between exosome-based vaccines and the gut microbiome is garnering increasing attention, given the microbiome’s pivotal role in immune modulation. The gut microbiome, a complex community of microorganisms residing in the gastrointestinal tract, has significant interactions with the host’s immune system [161]. Shifts in microbiome composition can impact systemic immune responses, including those triggered by vaccines [162]. Certain microbial populations, for instance, can amplify vaccine effectiveness by facilitating T-cell activation and antibody production. For instance, in preclinical studies, mice treated with *Akkermansia muciniphila* or Lactobacillus species showed increased responses to vaccines against viruses like the flu and polio. These bacteria are believed to produce metabolites that promote the maturation and activity of dendritic cells, which are key in initiating T-cell responses, thereby amplifying the overall immune response [163]. A notable example is the Bifidobacterium species in the gut microbiome, which has been shown to enhance the efficacy of vaccines. In studies involving influenza vaccination, the presence of Bifidobacterium was associated with increased levels of vaccine-specific antibodies and a more robust T-cell response [164]. Conversely, dysbiosis, a disruption in the microbiome’s balance, may impair immune functionality and reduce vaccine efficacy. The gut microbiota and the immune system maintain a dynamic, symbiotic relationship [165,166]. Microbial metabolites, such as short-chain fatty acids (SCFAs), secondary bile acids (BAs), and inosine, tryptophan derivatives, and polyamines, produced by the gut microbiota, significantly impact immune responses and can enhance vaccine effectiveness [167]. SCFAs, for example, support T-cell activation and increase cytokines like IL-22 and IL-17, strengthening the gut barrier and immune readiness [168]. Bile acids and tryptophan metabolites interact with receptors such as Farnesoid X Receptor and Takeda G Protein-Coupled Receptor on immune cells, modulating inflammation and promoting immune balance, which are essential for an optimal vaccine response [169]. Inosine and polyamines further support immune-cell activation and resilience, enhancing the body’s ability to generate robust antibody and T-cell responses post-vaccination [170]. Together, these metabolites from a healthy gut microbiota create a supportive immune environment, potentially amplifying the effectiveness of vaccines.

The administration of milk exosomes as cancer vaccines could influence the gut microbiome in multiple ways [171]. These exosomes may introduce new substrates or signaling molecules that alter microbial growth and activity [49]. Moreover, the immune response generated by the exosome-based vaccine may create a microenvironment favoring beneficial microbes while suppressing harmful ones [150]. This immune modulation could, in turn, reshape the microbiome towards a more balanced and health-supportive state. Furthermore, the specific antigens delivered by milk exosomes could elicit tailored immune responses that interact with gut microbiota. A potent immune reaction might facilitate pathogen clearance, thereby promoting a favorable microbiome composition [172]. One of the most exciting applications of mEVs is their potential as platforms for oral vaccination. Their stability and resilience make them ideal for surviving the gastrointestinal tract and delivering antigens to gut-associated lymphoid tissues (GALT), inducing mucosal immunity. This property is particularly beneficial for developing vaccines against intestinal pathogens like Salmonella and rotavirus [173,174]. Bovine milk exosomes, for instance, have been shown to influence the gut microbiome composition in nonbovine species, hinting at a potential mechanism of interaction between dietary components and the gut immune system [170]. Such characteristics underscore the versatility of mEVs in oral vaccine formulations, which could improve patient compliance and have significant implications in low-resource settings.

**Figure 2:**
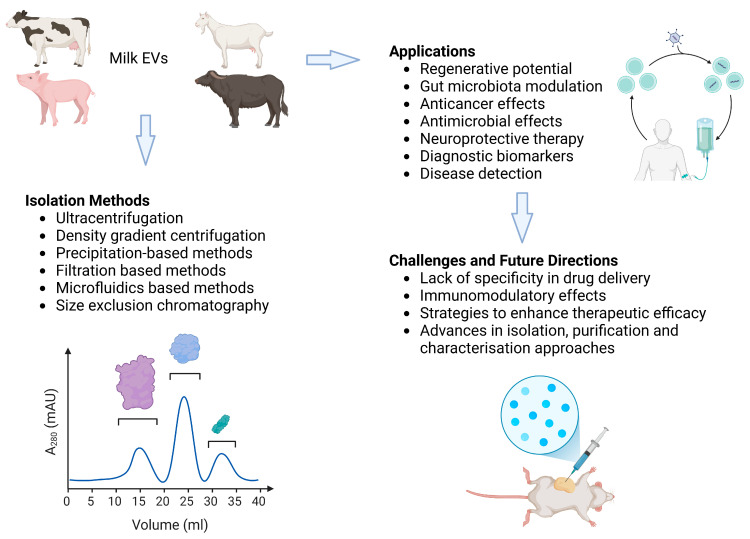
The overview of animal-derived EVs with isolation, characterization, therapeutic applications, challenges, and opportunities associated with EVs.

However, mEV-based vaccines face several challenges. Firstly, the complexity in cancer applications arises from mEVs’ dual roles as inducers of metastasis and suppressors of primary tumor growth, with contextual factors like the timing of administration significantly influencing their effects [175]. Additionally, ensuring the effective loading of antigens into mEVs and achieving consistent delivery can be challenging, necessitating the optimization of methods for isolating and characterizing mEVs to maintain their functional properties [176]. Regulatory considerations also play a critical role, as the use of mEVs in vaccines requires comprehensive safety and efficacy evaluations to meet stringent regulatory standards [174]. Moreover, a deeper understanding of the immunological mechanisms at play is essential to optimize mEV use in vaccine strategies, ultimately enhancing their efficacy and safety. Lastly, the scalability of mEV production poses logistical and economic challenges that must be addressed to ensure that mEV-based vaccines can be produced at a scale suitable for widespread public health applications [177]. Preclinical evidence suggests milk-derived exosomes are promising for therapeutic applications, particularly in immunomodulation, oxidative stress reduction, and the enhancement of cellular functions. Their biodistribution varies with administration routes, presenting opportunities for targeted therapies. Further research is required to optimize their use and fully elucidate their mechanisms of action. Figure 2 provides an overview of animal milk derived EVs and their isolation, characterization, therapeutic applications, and associated challenges and opportunities.

## 7. Conclusions

In conclusion, animal-derived EVs represent a burgeoning field of research with vast implications for various applications in medicine, agriculture, and beyond. mEVs, in particular, have garnered significant attention due to their abundance, stability, and diverse cargo of bioactive molecules. These vesicles, including exosomes, hold immense promise for therapeutic interventions, diagnostic utilities, targeted drug delivery, and vaccine development. The immunomodulatory properties of mEVs, driven by their miRNA content and active components, have been extensively studied across different mammalian species, showcasing their potential in regulating immune responses, inflammation, and tissue repair processes. mEVs’ ability to modulate immune cell function, promote immune tolerance, and serve as adjuvants suggests they could enhance vaccine efficacy by fine-tuning the immune system’s response. Furthermore, mEVs have demonstrated the ability to traverse physiological barriers, survive harsh conditions, and modulate gene expression in recipient cells, highlighting their suitability as delivery systems for both therapeutic agents and vaccines. Research has also uncovered their role in influencing various physiological processes, including bone metabolism, wound healing, and cancer progression. These vesicles possess intrinsic anticancer effects and can enhance the efficacy of chemotherapeutic agents when used as carriers. Additionally, mEVs have been implicated in regulating gut microbiota, promoting intestinal health, and mitigating inflammatory bowel diseases. However, challenges remain in optimizing the specificity, efficacy, and safety of mEV-based therapies, including their use in vaccine development. Strategies to enhance tissue targeting, cargo loading, and the scalability of production are essential for clinical translation. Comprehensive preclinical and clinical studies are also necessary to evaluate the long-term effects, biodistribution, and toxicity profiles of mEVs. Overall, the multifaceted properties of animal-derived EVs, particularly mEVs, offer exciting prospects for advancing biomedical research, therapeutic interventions, and vaccine innovations. Continued interdisciplinary collaboration, technological innovation, and translational efforts will be paramount in harnessing the full potential of mEVs for addressing diverse health challenges and improving both human and animal well-being.

## Figures and Tables

**Table 1:** Therapeutic potential of milk-derived EVs and their mechanisms of action and specific interactions within the body.

Function	Mechanisms of Action	Pathways Involved	Potential Applications	References
Immunomodulation	Modulate macrophage differentiation, cytokine secretion, and T-cell responses.	TGF-β signaling, NF-κB pathway	Immune therapy, prevention of autoimmune diseases	[70,71,72,73]
Modulation of Gut Microbiota	Promote growth of beneficial bacteria and enhance gut barrier integrity.	IL-10 signaling, MAPK pathway	Treatment of inflammatory bowel disease (IBD)	[74,75,76,77]
Anticancer Effect	Deliver chemotherapeutics and RNAs, enhancing stability and targeting to tumor cells.	PI3K/AKT pathway, apoptosis pathway	Cancer therapy, targeted drug delivery	[78,79,80,81,82,83,84,85,86,87,88,89,90,91,92,93,94]
Wound Healing	Promote cell proliferation, angiogenesis, and extracellular matrix formation.	TGF-β/Smad signaling, Wnt signaling	Tissue regeneration, chronic wound management	[95,96,97]
Inflammation	Enhance pro-inflammatory responses and modulate cytokine expression to improve intestinal barrier function.	NF-κB pathway, JAK/STAT pathway	Management of inflammatory conditions	[98,99,100,101,102]
Skin Health	Protect skin cells from UV damage, enhance collagen synthesis, and reduce melanin production.	MAPK pathway, PI3K/AKT pathway	Cosmetic applications, anti-aging treatments	[103,104]
Bone Regeneration	Influence osteogenesis and bone metabolism through bioactive components.	Wnt/β-catenin pathway, BMP signaling	Orthopedic applications, treatment of bone fractures	[105,106]
Hepatoprotective Effects	Reduce oxidative stress and inflammation; enhance liver cell survival.	Nrf2 pathway, PI3K/AKT pathway	Treatment of liver diseases, fatty liver disease	[107,108]
Antibacterial Effects	Inhibit bacterial growth and biofilm formation through the release of antimicrobial peptides and enzymes.	MAPK pathway, oxidative stress pathways	Treatment of bacterial infections	[92,109,110]
Antiviral Effects	Interfere with viral replication and entry; enhance host antiviral response through immunomodulation.	JAK/STAT signaling, NF-κB pathway	Treatment of viral infections, enhancement of vaccines	[111]

**Table 2:** Overview of different milk-derived extracellular vesicles (mEVs) and their therapeutic applications.

mEV Type	Used as Drug Carrier	Used as Treatment Alone
Cow mEVs	Paclitaxel-loaded mEXOs, doxorubicin-loaded mEXOs for targeted cancer therapy [82,84]	Modulation of gut microbiota, alleviation of inflammatory bowel disease [75,76]
Goat mEVs	Chlorin e6-loaded mEXOs for photodynamic cancer therapy [79]	Enhanced macrophage activation, inducing pro-inflammatory cytokines [90]
Buffalo mEVs	-	Potent antitumor effects in colon cancer cells [91]
Camel mEVs	-	Selective apoptosis in HepG2 and CaCo2 cells, sparing normal cells [92]
Yak mEVs	-	Improved hypoxia tolerance in intestinal cells under stress [93]
Colostrum-derived mEXOs	-	Highest apoptotic activity on HepaRG cells, anti-inflammatory and anti-angiogenic properties [94]

**Table 3:** The summary of targeting approaches focuses on mEV.

Targeting Strategy	Modification/Description	Therapeutic Outcome	References
Surface Modification with Folate	Folate-conjugated lipids attached to mEV surface	Targets folate receptor-positive tumor cells, enhancing doxorubicin delivery and drug uptake	[130]
Superparamagnetic Iron Oxide Nanoparticles (SPIONs)	Loading mEVs with SPIONs, enabling magnetic navigation with external magnetic fields	Improved tissue penetration, especially in poorly vascularized tumors	[131,132]
PEGylation	Polyethylene glycol (PEG) coating on mEVs	Enhances mucus penetration, stability, circulation time, and protects siRNA for oral delivery	[135]
Ligand Modification (Peptides/Antibodies)	Specific peptides or antibodies attached to mEV surface	Selective binding to overexpressed tumor cell receptors, improving targeted drug delivery	[136]
Hyaluronic Acid (HA) Coating	HA binds to CD44 receptors on tumor cells	Delivers miR-204-5p mimics, achieving antitumor effects with minimized side effects	[137]
Folate Receptor Targeting	Folate receptor-specific targeting, especially in NSCLC and lung adenocarcinoma	Enhanced delivery of anticancer agents like apherin A or paclitaxel	[138]
Hyaluronic Acid with Doxorubicin	HA conjugated to mEVs for selective targeting of doxorubicin	Enhanced therapeutic outcomes with reduced systemic toxicity	[139]
Tumor-Suppressive miRNAs	Bovine mEVs delivering miRNAs like miRNA-125b and miRNA-let-7	Inhibits oncogenic pathways in prostate cancer cells	[140]

## Data Availability

There are no data to support the findings of this review.
